# Chronic lead malposition diagnosis and management: discussion of two cases and literature review

**DOI:** 10.1002/ccr3.819

**Published:** 2017-02-01

**Authors:** Ahmed Almomani, Amjad Abualsuod, Hakan Paydak, Wilburt Peer, Waddah Maskoun

**Affiliations:** ^1^ Division of CardiologyUniversity of Arkansas for Medical SciencesLittle RockArkansasUSA; ^2^ Department of Internal MedicineUniversity of Arkansas for Medical SciencesLittle RockArkansasUSA; ^3^ Division of CardiologyCentral Arkansas Veterans Healthcare SystemLittle RockArkansasUSA

**Keywords:** 12‐lead ECG, lead extraction, lead malposition, ventricular tachycardia

## Abstract

Management of lead malposition is crucial to avoid complications and is carried out on case‐by‐case bases. The 12‐lead ECG during pacing and chest X‐ray are essential during initial workup and recommended for new patients to the device clinic. Echocardiography and CT scan are important to confirm the location and plan appropriate therapy.

## Case 1

**Figure 1:**
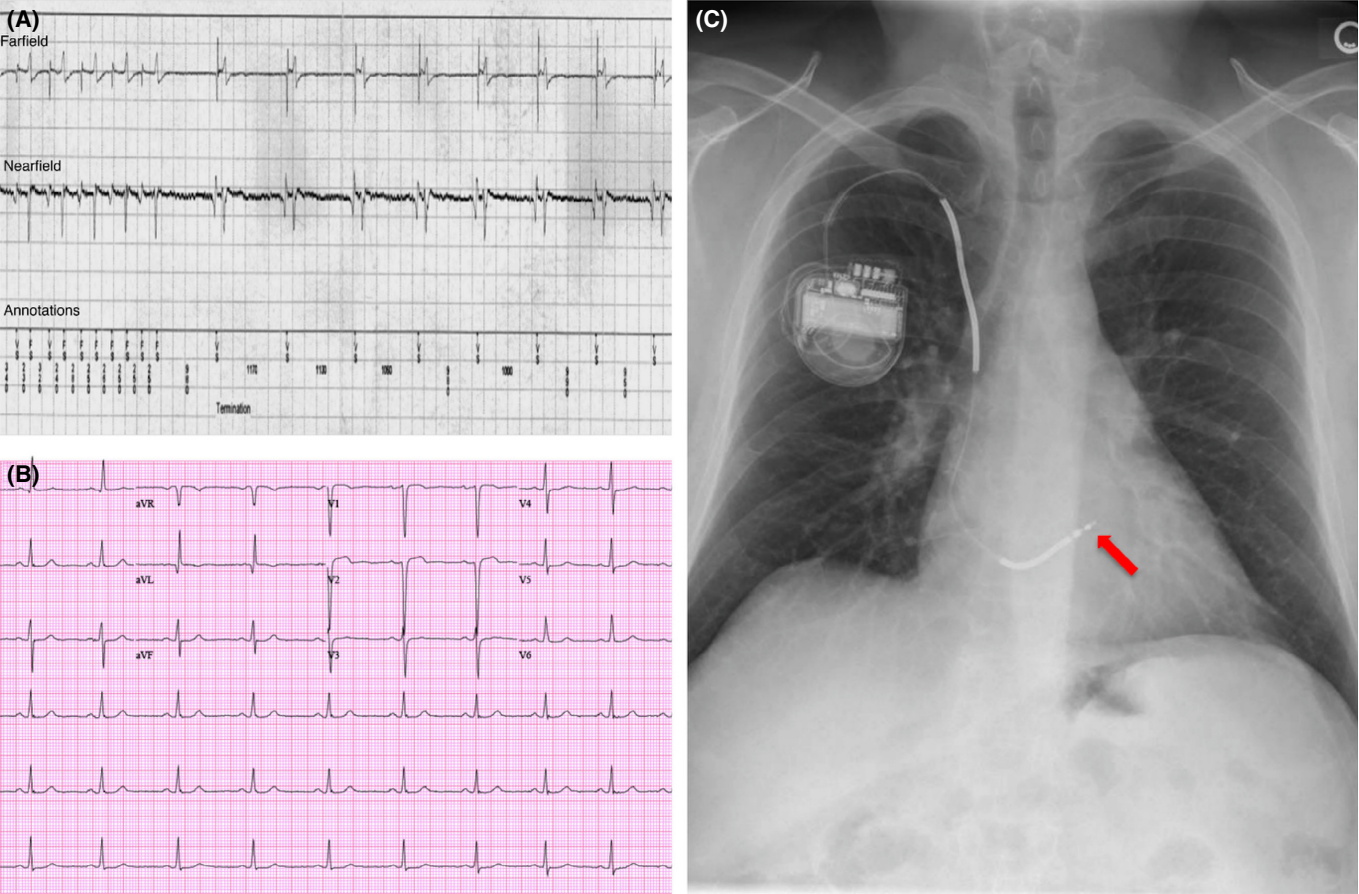
Example of the short V‐V intervals episodes detected on device check (A). 12‐lead EKG (B) and posterior–anterior projection chest X‐ray demonstrating ICD lead position (arrow) (C).

The patient was a 42‐year‐old male with coronary artery disease and ischemic cardiomyopathy who underwent right‐sided single‐chamber Medtronic Protecta implantable cardioverter defibrillator (ICD) (Lead Model: 6947M62 [Medtronic Inc., Minneapolis, MN]) placement for primary prevention by cardiothoracic surgery in August of 2012. Patient presented to our device clinic in June of 2014. He had no ICD shocks, and he leads a very active lifestyle. On device interrogation, he was found to have multiple asymptomatic tachycardia episodes. The device intracardiac electrograms (EMG) were reviewed and showed multiple runs of short V‐V tachycardia, which was interpreted as nonsustained ventricular tachycardia (NSVT) (Fig. 1A). These episodes were not related to activity, medication, or device interference, and they take place during anytime of the day/night. The 12‐lead EKG and chest X‐ray were not remarkable (Fig. 1B and C). However, when we compared the ventricular signal during the short V‐V interval seen in device interrogation to the ventricular signal during sinus rhythm, they looked similar except for further separation of the two components of the ventricular signal during the short V‐V interval on the EMG.

Differential diagnosis for this short V‐V interval included the following: (i) T wave oversensing 1; however, during normal sinus rhythm, the separation was too short and was not expected to change significantly during tachycardia and the V‐V interval was too short during tachycardia. (ii) Wide QRS with R wave double counting 2, which was not the case in our patient as shown in his baseline ECG and would not explain the separation of the two components of the ventricular signal during tachycardia. (iii) Fractured lead with oversensing 3, in which the near‐field EMG would look different. (iv) Far‐field P wave oversensing, which may happen in a patient who has integrated bipolar lead with close proximity of the RV coil to RA or the tip of the RV lead, is close to the AV annulus. In cases of P wave oversensing, the A and V are expected to be further apart during atrial tachycardia similar to what our patient had. However, the A and V components were shorter than expected during sinus rhythm in our patient, but it was still a possibility.

**Figure 2:**
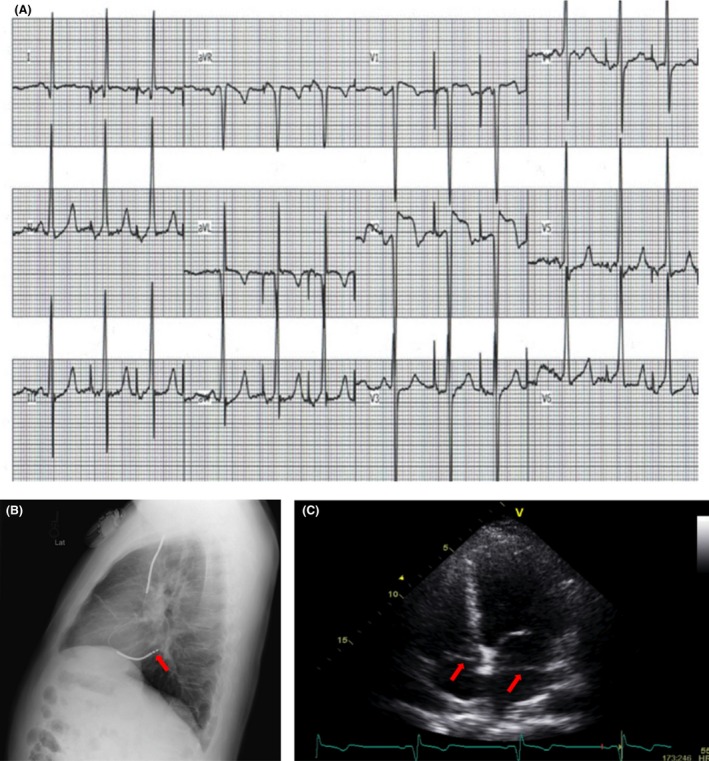
Pacing at maximum output with left atrial capture. The first beat was not paced with normal sinus beat morphology (A). Chest X‐ray with lateral projection demonstrating ICD lead position (arrow) suggestive for misplacement within the coronary sinus or left atrium (B). 4‐chamber view of transthoracic echocardiography showing the ICD lead creating a shadow in the left atrium (arrow) (C).

We tried pacing the ICD lead at the maximum output as part of workup to evaluate the possible diagnosis, which resulted in atrial capture only and morphology of the P wave was consistent with left atrial capture (Fig. 2A) and confirmed lead misplacement. Chest X‐ray with lateral projection (Fig. 2B) was obtained and demonstrated ICD lead malposition into the coronary sinus (CS) or left atrium. Echocardiography was performed and confirmed lead misplacement in the CS and not in the left atrium. (Fig. 2C). The two components were basically both near‐field A and V (not far field) and the episodes reported as NSVT were actually atrial tachycardia episodes with further separation of the A and V due to the AV nodal delay during the tachycardia. The fact that the tachycardia episodes terminated with V each time ruled out ventricular tachycardia with one‐to‐one retrograde conduction.

Due to the young age of the patient and higher risk of complication if lead extraction is to be needed in the future, we elected to attempt lead removal and repositioning instead of just abandoning that lead.

Lead revision was done in July of 2014 (23 months after implantation) under fluoroscopy guidance in the operating room due to concern about possible perforation and pericardial effusion with the lead removal from the CS. The lead was removed from the header, and a stylet was placed. After unscrewing the lead and with gentle traction, the lead came out without any difficulty. The lead was then positioned in the right ventricle apex and was screwed in with no complications and good pacing numbers. Transthoracic echocardiogram was obtained, and no pericardial effusion was detected.

Patient was seen during follow‐up at 1 and 6 months, and ICD interrogation showed normal sensing, pacing, and no more episodes of short V‐V tachycardia.

## Case 2

**Figure 3:**
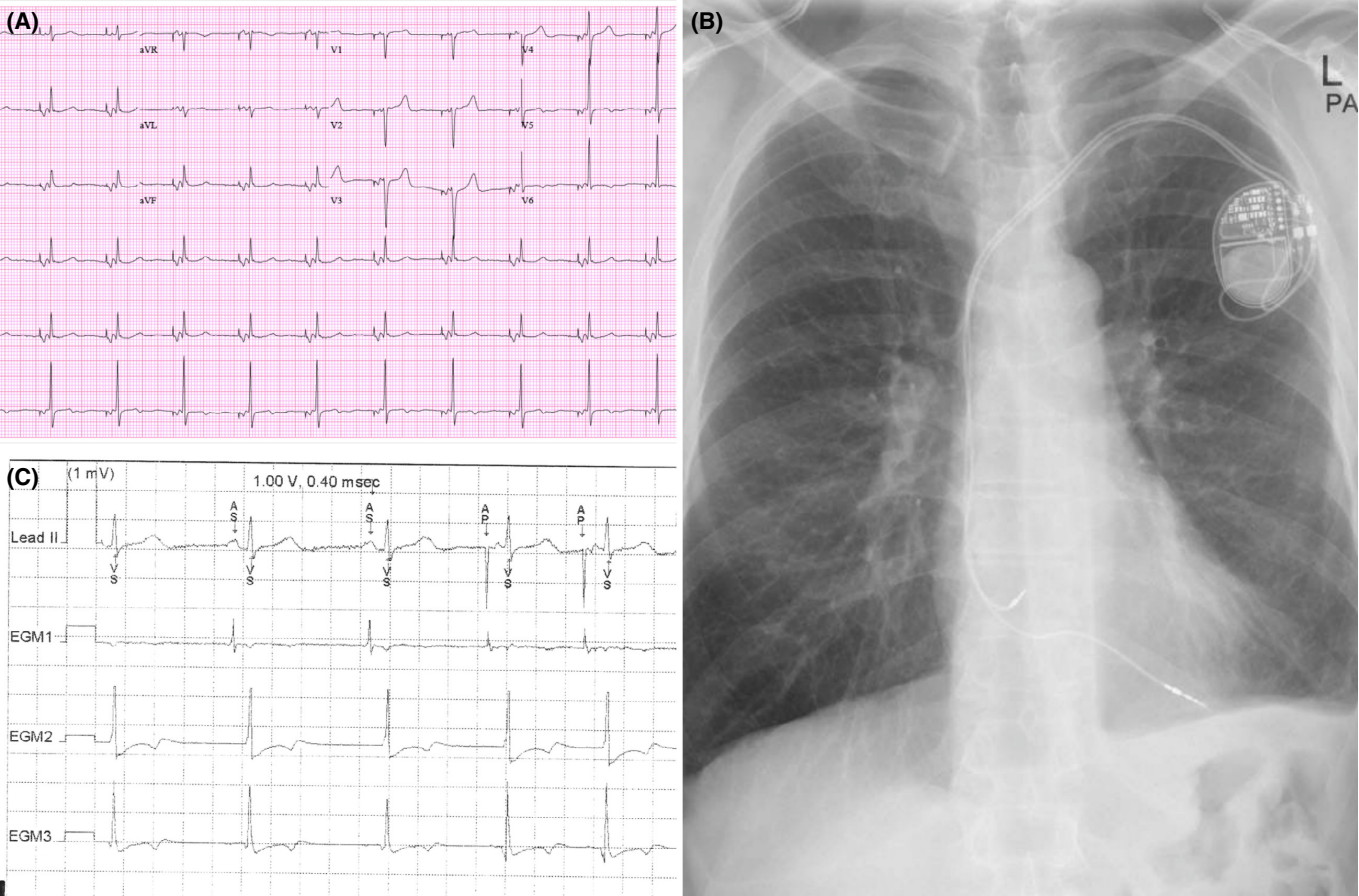
EKG with paced P wave axes and morphology with left atrial pacing (A). PA Chest X‐ray (B). Device interrogation and threshold testing (C).

The patient was a 63‐year‐old male with symptomatic bradycardia who underwent dual chamber pacemaker (Medtronic Advisa DR MRI A2DR01) in February of 2006 by cardiothoracic surgery. Patient was referred to our EP clinic for generator change due to ERI on device check 9 years later. Patient reported no complaints and has been asymptomatic. He has past medical history significant for diabetes and hypertension. On review of the EKG, P wave axes and morphology were abnormal and consistent with left atrial pacing (Fig. 3A). PA chest X‐ray was not remarkable (Fig. 3B).

**Figure 4:**
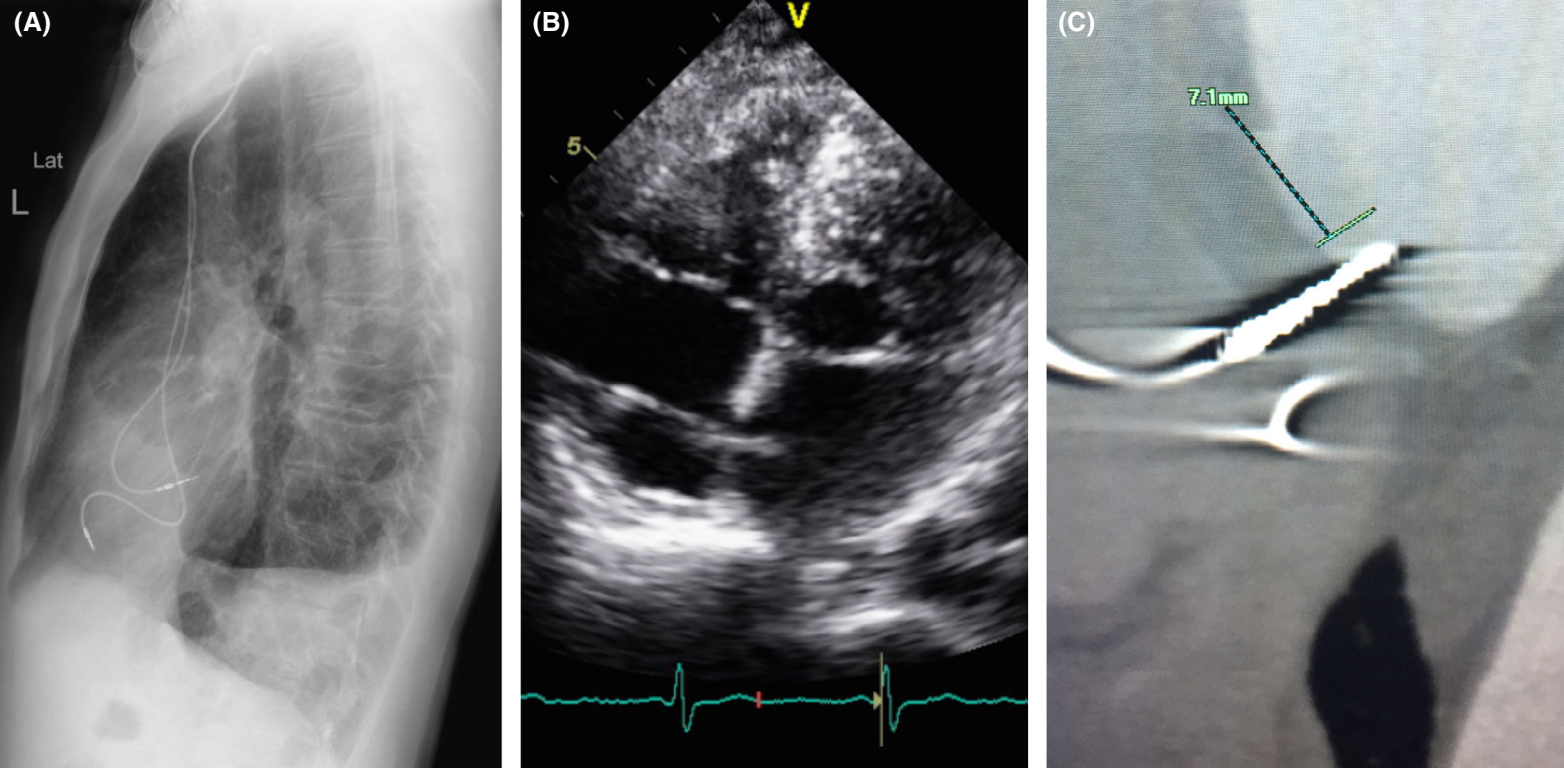
Previous lateral chest X‐ray demonstrating that the atrial lead was more posterior in position that expected for an RA lead (A). Transthoracic echo subcostal view demonstrating lead crossing the intra‐atrial septum to the LA (B). Cardiac CT scan with reported 7.1 mm of the lead is protruding into the left atrium (C).

On device interrogation, one component was mainly present in the atrial lead EMG, (Fig. 3C) which raised the suspicion that the atrial lead (Medtronic 5076 CapSureFix Novus) is not in the AV annulus and the possible malposition in the left atrium. On review of a previous lateral CXR, the lead was more posterior in position than expected for an RA lead (Fig. 4A). Echo was obtained and was suggestive of lead crossing the intra‐atrial septum to the LA, (Fig. 4B) which was confirmed with a cardiac CT scan that showed 7.1 mm of the lead was protruding into the left atrium (Fig. 4C). Bipolar pacing only captured the atrium even at maximum output.

In an attempt to determine whether the atrial lead was in direct contact with the atrial wall or whether it was an unusual type of anodal capture with the lead ring, and because it protruded only 7.1 mm into the left atrium and was still capturing, we tried tip to ring (bipolar) pacing versus tip to can (unipolar) pacing. There was left atrial capture with the unipolar pacing as well, and it was with a lower threshold compared to bipolar (0.5 V @ 0.5 msec versus 0.75 V @ 0.5 msec) making the possibility of anodal capture with the lead ring unlikely. This could have been an indication that the atrial lead tip was actually in direct contact with, or at least very close to, the left atrial wall, which a good‐quality CT scan failed to show. The second possibility is that there was noncontact capture, which to the best of our knowledge has not been reported before. However, higher output to capture will be expected.

As mentioned above, the patient denied any symptoms of CVA or TIA since device implant 9 years before, which created a long discussion with the patient about whether he would consider anticoagulation, especially because only a small portion of the atrial lead was present in the left atrium. Ultimately, we elected to start the patient on oral anticoagulation, and he was agreeable with that.

## Discussion

Cardiac device therapy is being used increasingly to treat heart rhythm disorders. Transvenous implantation of a pacemaker or implantable cardioverter defibrillator lead into the left atrium or ventricle is a rare complication of cardiac pacing 4. The pacemaker lead may enter the left atrium through a patent foramen ovale, a sinus venosus, an ostium primum, or an ostium secundum atrial septal defect 5. It may also happen when screwing the lead through an intact fossa ovalis using a stylet, especially when only a small portion of the lead is noted in the left atrium. The lead can also directly enter into the left ventricle via the aorta by accidental puncture of the subclavian artery or due to an interventricular septal defect 6.

Patients with lead malposition in the left heart chamber may be asymptomatic or may present with complications secondary to arterial embolization. The traditional management has mainly focused on systemic anticoagulation with warfarin in order to minimize TE complications. The use of antiplatelet therapy as a sole means of preventing TE complications is not advisable 7, 8. It has been reported that the incidence of thromboembolic events is about 37% in patients with lead misplacement in the left cavities and not receiving anticoagulation; almost all leads in these reports were in the left ventricle 8, 9. Currently ongoing clinical trial, ALternate Site Cardiac ReSYNChronization (ALSYNC) Study, aims to evaluate the safety and performance of the investigational atrial transseptal endocardial LV lead delivery system and the implant procedure for delivering the lead into the left ventricle via a superior approach, and to evaluate the performance of the lead in the left ventricle. Initial data showed that of 118 successful left ventricular lead implantations, which received effective anticoagulation (warfarin with target INR range 2.5–3.5), five patients had cerebrovascular accidents none of which led to permanent disability as defined by Rankin class 3 or greater. In a preclinical safety study carried out prior to starting the ALSYNC trial on animal models, only the animals in the warfarin group were free of any small infarcts in the kidneys at 2 years 10.

The type and material of the lead can affect thrombogenicity 11, 12. Although this has been demonstrated only in small animal studies, it may be taken into consideration for individualization of therapy especially if the lead material is known to be highly thrombogenic. The distal portion of the Medtronic 5076 CapSureFix Novus lead that our patient had was comprised of the electrode helix which is platinum covered by fine texture platinum black. The polymer section between the tip and ring electrode is polyether polyurethane, which is much less thrombogenic than the silicone rubber of the lead body that is proximal to the ring electrode 11, 12. The ring electrode material is the same as the helix tip. If only 7.1 mm is truly in the LA, then the platinized helix and polyurethane are the materials exposed.

The detection of an unusual arrhythmia pattern on EKG or device interrogation should always promote the workup of lead malposition, migration, perforation, or dysfunction 3, 5. EKG, in particular, is a useful diagnostic tool for detecting the position of a cardiac stimulation. Chest X‐ray with posterior–anterior and lateral views can provide supplementary diagnostic arguments, as was the case in our patients. Indeed, for a correctly placed lead into the right atrium and ventricle, a lateral chest X‐ray shows the anterior position of the lead and above the diaphragm. On the other hand, a lead located relatively high above the diaphragm in the posterior–anterior view and more posteriorly placed relative to the sternum in the lateral view should suggest misplacement/malposition into the left ventricle or CS 5, 13, 14. Two‐dimensional echocardiography is a valuable tool for the diagnosis of lead misplacement, perforation, migration, and dislodgement. Difficulty or failure of 2D TTE to visualize leads is not uncommon. Transthoracic real‐time 3D echocardiography is complementary to 2D echocardiography in detecting lead position and should be used if a lead complication is suspected 15. Cardiac CT plays a crucial role in the diagnosis of lead misplacement and perforation when other modalities are nondiagnostic 5, 14, 16. However, CT images may be limited by artifacts created by the leads.

Once the diagnosis of malposition is confirmed, the patient may simply be managed with anticoagulation or by properly reinserting the leads, considering the benefits and potential risks 8, 17. Subsequent removal of these systems with their leads may be necessary for various reasons. Although there is accumulating evidence in the literature to support the safe and successful percutaneous extraction of leads for pacemaker and ICD from the right side of the heart 18, this is currently not the case for left‐sided extraction. In the 2009 Heart Rhythm Society (HRS) expert statement, the removal of anomalously placed LV leads was a class III recommendation 19. The expert statement advised surgical removal with cardiopulmonary bypass in compelling clinical scenarios and cautioned against the percutaneous removal of left‐sided endocardial leads 19. However, few cases of percutaneous pacemaker and ICD lead extraction for leads accidently placed in the left ventricle have been reported, some of which had been in place for up to 8 years 20, 21. Embolization prevention devices have been used in the internal carotid and vertebral arteries in at least one reported case 22, but the literature is sparse in regard to left atrial lead extraction.

In our first case, the short V‐V tachycardia was actually an atrial tachycardia due to lead malposition. The pacing morphology of the P wave, the lateral X‐ray, and echocardiography were all very helpful to confirm the diagnosis of lead misplacement within the CS. Chest CT can help distinguish lead placement in the CS versus left atrium. However, based on the good image quality of the echocardiography in this patient, we did not feel that chest CT was indicated in his case.

Previous series of coronary sinus pacemaker lead extraction have been published. The largest study to date by Bongiorni et al. 23, reported on 37 patients. The longest CS lead had been in situ for 84 months. Seventy three percent of the leads were removed with manual traction and the remaining 27% were successfully removed using mechanical dilatation with polypropylene sheaths. No major complications were reported and their analysis did not identify preoperative markers that predicted the failure of manual traction. However, there is not much data on the safety of extracting an inadvertently placed ICD leads in the CS. In our case, the lead was an ICD lead implanted from the right side and was inadvertently placed and actively fixed within the CS 23 months ago. We were able to remove the lead without any difficulty and reposition it without immediate or late complications.

In the second case, lead misplacement was suspected from P wave morphology on surface EKG and confirmed with the CXR. Echocardiography was suggestive of lead in the left atrial and not the CS, but a CT scan was crucial to confirm the finding and measure the portion of the lead in the left atrium. We decided to anticoagulate the patient at the end even though he had no symptoms for 9 years.

## Conclusions

Management of leads with chronic misplacement/malposition to avoid inappropriate device therapy, thromboembolic events, and complications if extraction was considered is typically a challenge and should be carried out on a case‐by‐case basis and includes extraction, abandoning the lead, and/or anticoagulation. 12‐lead ECG during pacing and PA & LA Chest X‐ray are extremely helpful to suspect or to confirm the diagnosis of lead misplacement/malposition and perhaps they need to be done routinely for all new patients to the device clinic with implanted device besides the device interrogation. Echocardiography and/or CT scan are typically the next step to confirm the location of the leads.

## Authorship

AA: wrote the main manuscript. AA: helped writing the manuscript. HP: revised the manuscript. WP: participated in optimization of images. WM: revised the manuscript and obtained the consent from patients and clinical care of both cases.

## Conflict of Interest

None of the authors has any conflicts of interest.
